# The Brazilian dental science

**DOI:** 10.1590/2176-9451.19.2.014-014.edt

**Published:** 2014

**Authors:** David Normando

**Affiliations:** Editor-in-chief (davidnormando@hotmail.com)

"Men stand out for what they do; women, for what they induce men to do".

(Carlos Drummond de Andrade, Brazilian poet from Minas Gerais state) 

Three years ago, a sublime editorial^1^ praised the audacious growth of Brazilian dental scientific production. At that time, our former editor dared to foresee the position Brazil would occupy in 2015: the most productive country in terms of dental scientific knowledge. The author was based on data from Scopus which, at that point, did not include *Dental Press Journal of Orthodontics* (DPJO). This database currently provides data as far as 2012, with DPJO having the highest number of scientific publications in Brazilian Dentistry.

In the current scenario, as far as 2012, Brazil still occupies the second position in world scientific production. However, we have begun to peep, as a lynx, the fact that we have come nearer the United States, the most productive country in terms of scientific production. We are growing like a giant. In 2009, for instance, the American dental scientific production was 66% greater than the Brazilian production. Nevertheless, this difference narrowed down to 20% in 2012. We should not be surprised if Brazilian scientific production reaches the first position yet in 2014, thus, corresponding to what had been wisely foreseen.^1^ Moreover, we are certain that indexing DPJO into Scopus has contributed to accelerate such progress.

A reasonable amount of self-contemplation would reveal even more flaring indicators. Twelve years ago, in 2002, Scopus registered only 171 articles published by Brazilian authors within the field of Dentistry. In 2012, there were 1,533. Our h-index - an index that measures the impact of the scientific knowledge produced by a country, university or author - has increased exponentially. Brazil currently occupies the eighth position in the Dentistry field, which is something for Brazilian scientific production, although still young, to be proud of. Despite any criticism, improvements in this index are strongly related to government policies of closely following the scientific production of Brazilian postgraduate courses. In other words, it all resembles a conductor who perfectly leads the drum section of a samba school playing in a constant rhythm.

Furthermore, made-in-Brazil journals have significantly contributed to highlight the notorious development of Brazilian postgraduate intellectual production. Brazil currently has seven journals indexed in Scopus: In 2012, these scientific journals published 675 articles (whereas there were only two journals in 2002, with 96 articles published). At this point, you may have squinted towards a major criticism: it is only numbers. But no. Given the substantial increase in Brazilian h-index and the healthy improvements in postgraduate scientific production, this editorial is not able to list all techniques that could be employed to correct such 'strabismus'.

In this context, Orthodontics has a lot to celebrate. In 2012, Brazil was the most productive country in terms of orthodontic publications, with DPJO - a journal indexed in the most important databases in the world - playing a major role. The h-index for Brazilian Orthodontics is the third in the world. We are certain that indexation of DPJO in PubMed/MEDLINE in 2013 will greatly contribute to future achievements.

Brazilian dental science is still simmering and, in a mathematical counterpoint, challenges the regression towards the mean. The position it has reached is a consequence of an action that involves an immeasurable number of researchers from the most secluded places of Brazil - who work overnight with passion and dedication; insane people who believe they can produce state-of-the-art scientific knowledge. Believing is a prime virtue, and that explains all the euphoria.

We have been conducted through this path by a beautiful and powerful leadership (as of a maestra): with indefatigable dedication and exhilarating inspiration - as a contagious samba that spreads itself among carnival revelers due to its sagacity. Only Drummond, in his sensibility, could perfectly describe the women of his homestate.
